# Persistent Pulmonary Hypertension: A Look Into the Future Therapy

**DOI:** 10.7759/cureus.20377

**Published:** 2021-12-13

**Authors:** Mridul Soni, Pranay K Joshi, Saawan C Patel, Devarashetty Shreya, Diana I Zamora, Gautami S Patel, Idan Grossmann, Kevin Rodriguez, Ibrahim Sange

**Affiliations:** 1 Research, Shri Lal Bahadur Shastri Government Medical College, Mandi, IND; 2 Medicine, B.J. Medical College, Ahmedabad, IND; 3 Internal Medicine, Pramukhswami Medical College, Karamsad, IND; 4 Research, Gandhi Medical College and Hospital, Secunderabad, Secunderabad, IND; 5 General Medicine, Universidad de Ciencias Médicas Andrés Vesalio Guzman, San José, CRI; 6 Research, Faculty of Medical Sciences in Katowice, Medical University of Silesia, Katowice, POL; 7 Research, Facultad de Medicina, Universidad Americana (UAM), Managua, NIC; 8 Research, K. J. Somaiya Medical College, Mumbai, IND

**Keywords:** new researches, newborn health, pulmonary critical care, treatment choices, high risk newborn, persistent pulmonary hypertension of the newborn

## Abstract

Persistent pulmonary hypertension (PPHN) of the newborn is a lung parenchymal disorder that causes a wide range of hemodynamic changes in the newborn's systemic circulation. Arising from a multifactorial web of etiology, PPHN is one of the most common reasons for neonatal intensive care unit hospitalization and is associated with increased morbidity and mortality. Historically, multiple treatment modalities have been explored, ranging from oxygen and surfactant therapy to newer upcoming medications like magnesium sulfate and adenosine. This review article has discussed the pathogenesis of PPHN and its relationship with the clinical implications of PPHN, such as heart failure and so on. This article has also explored the diagnostic guidelines and analyzed the existing and the upcoming modalities for treating PPHN.

## Introduction and background

Persistent pulmonary hypertension (PPHN) is defined as a cardiopulmonary disease marked by a high pulmonary arterial vascular resistance and extended right ventricle afterload stress [1]. Even though better obstetric care has decreased the frequency of these prenatal diseases, this remains a significant medical issue. Estimates suggest that up to 4% of all hospitalizations to the particular specialized neonatal intensive care unit are due to PPHN. In industrialized nations, the frequency varies from one to two PPHN every 1,000 live births, resulting death rate approaching 10% [2,3]. The data from the United States show prevalence for PPHN ranging from 0.4 to 6.8 for every 1,000 live births, whereas the United Kingdom shows fluctuation from 0.43 to 6 for every day 1,000 live births [4,5]. Despite improvements for PPHN treatment, newborns with mild to severe illness have a 10% premature death risk, which is much greater for newborns with pulmonary hypoplasia or congenital diaphragmatic hernia (CDH). PPHN has been linked with severe long-standing morbidities, involving up to 25% of babies having PPHN showing major cognitive defects by the age of two years [6-8]. Although improved maternity healthcare has significantly decreased the overall frequency of such prenatal diseases, PPHN remains a significant healthcare issue, consisting of around 7% of all hospital admissions to certain specialized medical wards [9]. PPHN is of two types, the acute and chronic types, with the sensitive type showing two more subtypes of reversible and irreversible [1].

Following are some of the critical factors linked with PPHN - cesarean section deliveries, maternal diabetes mellitus, and a high body mass index. Its pathology has been better appreciated, and many molecular pathways involved in pulmonary vascular tone control have been discovered. While some cross-connections and correlations are multidimensional, the routes could be categorized into the following groups for simplification: (A) nitric oxide (NO) soluble guanylate cyclase; (B) prostaglandin prostacyclin cyclic adenosine monophosphate (cAMP); (C) rho-A/rho-kinase; (D) endothelin; (E) free radicals [7-11]. The most helpful diagnostic technique is echocardiography, considered the gold standard for verifying PPHN [1]. Sustaining newborns are more likely to need cardiorespiratory care, have lengthy hospitalization, or show a greater risk of prolonged neurological problems [6]. This article aims to help better understand the therapeutic and management process in a more comprehensive way. It also helps us understand how the various treatment modalities are used, the standard interventions, and the importance of echocardiography in speeding up the diagnosis.

## Review

Pathophysiology

The placental membrane serves more than just a gaseous interchanging unit [12]. Due to the decreased pulmonary blood flow and higher pulmonary vascular impedance, approximately 13%-21% of the ventricle blood enters the fetus [13]. An amalgamation of alveolar bronchoconstriction and the omnipresence of essential bronchoconstrictor plays a significant role in maintaining an elevated pulmonary vascular resistance (PVR) during fetal life [14]. The physiological parameters such as establishing low-resistance placental perfusion, an upsurge in the systemic arterial tension, and an elevation in the blood circulation tend to occur immediately after birth. They are essentially crucial to mark a momentous fall [8]. A rise in the gaseous exchange and an increase in the blood circulation through both the lungs aids the dilatation of the bronchial system [8]. The intensification of the proper atrial tension and the closure of patent foramen ovale tend to be 8-10 times more than that of the fetal circulation [15]. The augmentation in the right atrium causes a spike in the oxygenation of the arteries, preceded by the closing of the patent ductus arteriosus and venous [15]. Consequentially, the fall in the PVR switches the blood circulation in the patent ductus arteriosus, followed by a fast respiratory capillary network [15]. The strain from heightened pulmonary perfusion and saturation precipitates endothelial NO release inside the airways and plays an imperative role in the cardiovascular shift post-delivery [16]. The arachidonic acid and prostacyclin mechanisms initiate adenylate cyclase and augment cyclic adenosine monophosphate within the smooth muscles, which necessitates an increase in respiratory dilatation [16]. Incapacitation in establishing the circulatory shift during delivery causes PPHN among newborns, which coerces the need to distinguish it from hypoxemia due to a high pulmonary resistance [11]. PPHN can be attributed to erroneously adapting pulmonary structure, which could be strongly affiliated with parenchymal illnesses of the respiratory systems such as meconium aspiration, respiratory distress syndrome, infections occurring in the lungs, or the ubiquity of widespread sepsis [11]. Although an elevated PVR could represent itself as a sole anomaly, sure newborns tend to present with concomitant fatalities, leading to a destructive cycle of hypoxemia, ventilation and perfusion mismatch, and cardiac inability to perform in its prime condition, precipitating widespread hypoperfusion and shock [11]. The establishment of a right-to-left shunt due to a constant patent ductus arteriosus predisposes to the unloading of lung circulation to avoid the collapse of the right ventricle resulting in global hypoperfusion [11]. Over the decades, biological mechanisms underlying PVR have become vital to physicians as they provide pertinent information regarding certain pathophysiological developments and clinical impacts. Such means include regulators of respiratory vasodilatation, which have been conventionalized to prioritize appropriate management in patients with PPHN [11]. Although the human body suffices innumerable commodious mechanisms, the ones most relevant to PPHN have been summarized in Table 1 [11].

**Table 1:** Mechanisms involved in persistent pulmonary hypertension

Mechanisms
Nitric oxide-soluble and Guanylate cyclase
Prostaglandin, Prostacyclin, and Cyclic adenosine
Rho-kinase
Endothelin
Free radicals pathways

Additional regulators have now been discovered, advancing the creation of alternate medication and enhancing the treatment modalities in patients presenting with the disease [17]. Inadvertent of the origination or molecular mechanisms, an elevated PVR insinuates to be the defining clinical trait for PPHN itself [18]. As the physiological and therapeutic implications tend to vary across different individuals, it becomes increasingly essential to note that the disease presentation could most ardently depend upon the presence or an absence of fetal shunts along with the heart's ability to withstand difficult situations [18]. Hemodynamic disruptions such as decreased pulmonary perfusion, hypoxemia, acidosis, ventilation-perfusion mismatch, and cardiac dysfunctions differ widely among patients depending on the severity, length of the original issue, and the root of the problem [19]. Compelling pulmonary vasoconstriction may precipitate an astringent ventilation-perfusion mismatch culminating in an inevitability of hypoxic respiratory failure [19]. The hypoxia may exacerbate, resulting in an additional decrease in pulmonary perfusion [19]. The presence of birth asphyxia further aggravates the decline in proper ventricular function [20]. The ventricular failure intensifies the hypoxia and systemic acidotic situation, causing exasperation of pulmonary arteries constriction, decreasing the effectiveness of respiratory vasodilatation therapy [20]. The persistently heightened PVR reduces left ventricular preload, pressuring the left ventricle to adjust through an elevated heart rate and force of contraction to maintain a sufficient amount of circulating blood flow, thereby establishing a compensation attained at the price of an increased myocardial usage [21]. Right ventricular enlargement induces the inter-ventricular membrane to deviate toward the left, provoking a massive decline intolerance and left ventricular filling volume itself [20]. The presence of a sizable patent ductus arteriosus shunting the blood from pulmonary to the systemic circulation might appear to be a paradox as it could safeguard the newborn's right ventricle by unloading the elevated pulmonary loop and supporting post-ductal systemic flow on the one hand while worsening the hypoxemia on the other [22]. The benefits and risks of fetal channels require careful analysis and considerations since a failure to address the presentation of PPHN might lead to refractory hypoxemia, which can endanger the patient by gravitating fatalities of systemic hypoperfusion and shock [23].

Risk factors

PPHN remains an alarming reason for newborn complications and mortality. Due to the failure of effective prevention or a cure for PPHN, early detection of the essential variables that cause PPHN is very important for a clinician [10]. Children delivered through a cesarean section with no preceding labor are at the most significant risk for PPHN [10]. Other essential variables linked to a higher risk of PPHN include high maternal body mass index, maternal diabetes, and CDH [10]. According to a study conducted in 2015, meconium is one of the main risk factors leading to blockage and gas trapping and it activates surfactants and hypoxia, causing pulmonary vasoconstriction [24]. A prospective data-collection study conducted in 1996 explained the prevalence of PPHN in patients with a CDH, concluding that the disease affects around one in every 2,000-3,000 live births [24]. A meta-analysis that was strategized from 1980 to 2008 revealed a decline in the quantity of alveolar, acinar, and artery cells in patients with PPHN, with a significant reduction seen in patients with CDH [25]. To contemplate the results of both the studies, infants suffering from CDH have been shown to possess a 60% mortality rate, with PPHN being the primary cause of death [25,26]. The newborn delivery mode tends to be a crucial risk factor for increasing the probability of the occurrence of PPHN [27,28]. A case-controlled study was conducted at the Madigan Army Medical Centre from 2003 to 2009, which identified 20 cases of primary PPHN. The odds ratio seen in that research was 4.9, which concluded that infants born through the caesarian section tend to have 4.9 times greater chances of developing PPHN than customarily paid infants [10,29].

Furthermore, a meta-analysis study conducted in Massachusetts in 2006 concluded that caesarian section, maternal ethnicity such as African and Asian population, and a high body mass index raised the risk of developing PPHN in the infants themselves [29]. Maternal medications such as aspirin and non-steroidal inflammatory drugs (NSAIDs), selective serotonin receptor inhibitors (SSRIs), and nicotine have been shown to pose as a crucial risk factor for the development of PPHN in newborns [30]. Aspirin and NSAIDs, which inhibit prostaglandin and thromboxane production, cause premature closure of the patent ductus arteriosus, leading to accelerated stress and increased blood pressure inside the pulmonary arteries [30]. According to a case-control study carried out in two critical care units, at Harvard, from 1985 to 1989, it was concluded that in comparison to the infants from the control group, the maternal usage of aspirin and NSAIDs increased the risk of PPHN development by five and six times, respectively [31,32]. In addition, based on the recently conducted case-control studies, the prevalence of PPHN seems to have increased by six times the standard value in mothers using SSRIs, with most of the evaluated results getting complicated due to an increasing frequency of parental depressive episodes in reproductive years [33,34]. Another case-control research proved that 14 out of 20 children with PPHN were exposed to SSRIs using in-utero [33]. A strong correlation for PPHN has been seen in mothers involved in smoking or nicotine abuse during the pregnancy [35]. Data analysis from Children's Hospital Oakland in 1992 revealed that 64.5% of babies with PPHN showed trace amounts of nicotine by-products in the umbilical cord blood (Table 2) [36].

**Table 2:** Risk factors for persistent pulmonary hypertension

Risk factors
Associated lung and heart disease: Congenital cystic, Adenomatous malformation, Alveolar capillary dysplasia, Pulmonary hypoplasia, Congenital heart defects, In utero ductus arteriosus closure, Congenital diaphragmatic hernia
Perinatal factors: Post maturity, Non-vertex presentation, Fetal distress, Cesarean section, Asphyxia, Twin-twin transfusion, Placental abruption, Intrauterine growth restriction
Postnatal factors: Sepsis, Inflammation, Oxidative stress
Antenatal drug exposure: Non-steroidal anti-inflammatory drugs, Selective serotonin reuptake inhibitors, Cigarette smoking
Maternal health status: Body mass index, Asthma, Diabetes mellitus, Urinary tract infection, Preeclampsia
Race and gender: African, Asian, Male

Diagnosis

PPHN among newborns presents with numerous symptoms that are not particularly distinctive to the condition. These symptoms may occur in various disorders other than PPHN, making a precipitant and precise diagnosis critical for the early identification of effective care of the disease [37]. About a suspicious patient history with lucid risk factors, PPHN is traditionally anticipated in newborns who show airway obstruction and heart disease during the first few hours of birth [37]. A rigorous investigation involving the appraisal of the clinical symptoms, evaluation of the entire case background, and a detailed physical examination may prove beneficial in revealing critical clues to the diagnosis [37]. Clinical assessments must be fast and conducted alongside resuscitative procedures, which mainly include continuous SpO_2_ monitoring, screening for oxygen status in the lungs, and stabilizing the vitals [37]. History of respiratory distress in the fetus, acidotic arterial blood gas, decreased Apgar scores, presence of meconium in the amniotic fluid, and chest X-radiation results are vital indicators for establishing the diagnosis of PPHN [37]. Blood, urine, or cerebrospinal fluid tests may show indications of acute systemic inflammation in addition to the clinical characteristics of septic shock or the presence of bronchopneumonia on a chest radiograph [37].

Moreover, echocardiography is considered one of the aptest bedside procedures performed for establishing the diagnosis, instituting an evaluation, and recording the severity of the PPHN [38]. A peak pulmonary pressure of greater than 35 millimeters of mercury supplemented by increased tricuspid regurgitation velocity, right to left shunts, and paradoxical septal movement at the end of systole can confirm the presence of of of PPHN [39]. Since the lung pressures are predicted to be high at birth and after that drop under physiological settings, this drop in the resistance may benefit babies with late-onset [40,41].

Management

With a better understanding of its progression, severity, and complications, the treatment of newborns with PPHN has improved. Various modalities cover an early recognition, alleviate the risk factors, and decrease the overall mortality associated with the disease [42]. The timely management of PPHN, which includes establishing a precipitant diagnosis identification of the fundamental causes and treatment of the underlying pathology, plays a significant role in modifying the severity of the disease [42]. Rapid recognition of signs and symptoms, immediate resuscitation, vigilant supervision, and proper progression of cardiovascular and respiratory treatments tend to be critical for managing PPHN [42]. The purpose of a circulatory evaluation becomes necessary as it ensures the adequacy of systemic perfusion along with the appropriate titration of the medications [42]. Hyperventilation, alkali infusion, sedation, and tolazoline have been the mainstays of therapeutic interventions before the introduction of inhaled NO, as evidenced by a review between 1993 and 1994 [42]. Hyperventilation poses a crucial management technique as it incites lung vasodilation by lowering blood carbon dioxide levels and elevating potential hydrogen, thereby reducing the systemic vascular resistance and curbing the elevated blood pressure [42]. The main underlying principle behind management is improving oxygenation, which can be achieved through administering oxygen, surfactants, vasodilator medications, and extracorporeal membrane oxygenation (ECMO) in refractory cases [42].

Surfactant

Surfactant shortage or deactivation has been linked with numerous neonatal pulmonary disorders such as respiratory distress syndrome, meconium aspiration, and pneumonia [43]. Immediate surfactant application and good pulmonary mobilization are linked to better results, lower risks, and reduced mortality in babies having parenchymal lung illness [43]. A multicenter, randomization, clinical, parallel-group study was undertaken in 13 tier III newborn critical care facilities within Germany during April 2009 and March 2012 [44]. The study had a patient size of 212 infants 50% of the subjects were given surfactants, and the other 50% were not; the survival rate in the surfactant population was 67.3% compared to 58.7% in the non-surfactant population, which concluded that surfactants increased the survival rates in PPHN. Surfactant administration permits the young child to keep breathing and utilize its larynx's physiological activities without needing an endotracheal tube [45]. Surfactant spreads fast and has been demonstrated in randomized controlled trials to lessen the requirement for respiratory support by leveraging its distinctive biophysical features without using a pressurized ventilator [45,46]. In a cohort study investigating 328 newborns affected with Meconium aspiration, septicemia, and PPHN, surfactant administration was linked to decreased oxygenation needs [47]. The research also suggested that using surfactants in the early stages of breathing minimized the need for extracorporeal pumping to manage breathlessness in term infants [47]. Surfactant has shown to decrease the intensity of respiratory illnesses as well as the proportion of infants with pulmonary complications requiring ECMO assistance post-birth [48].

Oxygen Therapy

A study conducted by Mandell et al. demonstrated that hypoxia triggers a vasoconstrictive reflex within the pulmonary vascular bed, which is an essential link in the physiological decline in PVR during parturition because of which the management has historically focused on maintaining above-normal oxygen levels while trying to manage newborns with PPHN [49]. In addition, research in the same paradigm showed that preceding hyperoxia caused enhanced pulmonary vasoconstriction following hypoxic shock, as well as a blunting of the vasodilatory effects of inhaled nitrous oxide (iNO) [49]. Furthermore, oxygen-free electrons have been reported to interfere with the nitrous oxide-producing molecules, linking them to increased pulmonary vascular constriction associated with right heart failure [50]. In infants with PPHN, preventing simultaneous hypoxia and hyperoxia while maintaining oxygen concentration within the normal physiological is considered the most appropriate therapeutic approach for the disease [50]. According to statistics, a PaO_2_ between 30 and 55 millimeters of mercury has enhanced lung dilatation in patients with bronchopulmonary dysplasia (BPD), establishing the presence of reduced alterations of vascular resistance in premature infants compared to term babies [51]. In newborns with PPHN, the best way to prevent hypoxia/hyperoxia is via diluting supplementary oxygenation to preserve concentration in the lower to mid-90s, including 90% and 97% warning thresholds. To conclude, this treatment modality demands the ubiquity of other robust data collection through randomized trials as a therapeutic intervention for PPHN [51].

Phosphodiesterase 5 Inhibitors

Being a potent phosphodiesterase-5 (PDE-5) inhibitor, sildenafil blocks cyclic guanosine monophosphate (cGMP) degradation and enhances arterial smooth muscle relaxation along with NO-facilitated vasodilatation [52]. According to a randomized control study conducted in Columbia by Baquero et al. on 25 patients with severe PPHN, 21/25 individuals showed massive improvement with Sildenafil administration in 25 hours itself [52]. Based on a study conducted in 2006 with a patient sample of 28 newborns, 14 in the control group and 14 in the trial group were administered with Sildenafil. Relapse developed in about 10 out of 14 control individuals; however, all sildenafil individuals could readily be weaned off without any deterioration [53].

Sildenafil has been shown to decrease mortality and severity of the disease, as evidenced by a meta-analysis conducted by Shah et al., comprising three randomized control trials that involved 77 patients diagnosed with PPHN [54]. The study highlighted the vital role played by sildenafil in bringing down morbidity by 95% in the majority of patients [54]. Another study conducted in 2020 that employed a randomized trial method revealed several beneficial therapeutic effects of using PDE-5 antagonists in individuals with PPHN compared to patients in the control group [55].

Phosphodiesterase 3 Inhibitors

Phosphodiesterase 3 (PDE-3) inhibitors increase the cyclic adenosine monophosphate levels, thereby enhancing the calcium influx within the cardiac myocytes resulting in a positive inotropic effect and rising heart contractility [56]. A study conducted by McNamara et al. at the Hospital for Sick Children in Canada from 2002 to 2004 studied a compiled case series of neonates with severe PPHN. It proved the effectiveness of Milrinone in enhancing pulmonary vasodilatation and decreasing the need for oxygen therapy in eight patients who previously failed to respond to inhaled nitrate therapy itself [56]. Similar therapeutic potential benefits of milrinone could be highlighted in the case of four newborns, as evidenced by a study conducted in Canada in 2004 [57]. A randomized, double-blind study conducted with 20 infants in Ireland has shown that the milrinone (50 micrograms per kilogram) loading dosage lasting one hour followed by two to three days of intravenous administration of 0.33 to 0.99 microgram per kilogram per minute substantially connected to increased respiration and even a reduction in iNO dose in case reports of 11 newborns with an adverse reaction to iNO [58].

Adenosine

Adenosine is a purine nucleoside that enhances sympathetic stimulation, promotes dilatation by increasing cAMP levels within vascular endothelial cells, activates potassium and adenosine triphosphatase pathways, and increments muscular tissue excitability [59]. Nine babies with chronic pulmonary hypertension requiring intensive care were investigated [59]. Six out of the nine infants having PPHN that required iNO for breathing assistance showed considerable improvement in response to oxygen delivery after a subsequent administration of a continuous infusion of 50 micrograms per kilogram per minute of adenosine [59]. According to research conducted in Australia in 1998 by Patole et al., five out of six infants with PPHN showed an upgraded response to oxygenation post-adenosine infusion therapy [60]. Adenosine is considered one of the most efficacious treatment modalities for PPHN in terms of ease of administration, early onset of action, a shorter half-life, and a predominantly safe therapeutic index [60]. The competency of the drug has been explained in a study conducted in the year 1996, which correlated the improvement in the partial pressure of oxygen of the newborns with PPHN after the administration of 50 micrograms per kilogram per minute adenosine over 24 hours [61].

Magnesium Sulfate

Magnesium is a vascular tone regulator that acts as a calcium blocker and requires the maintenance of concentrations to avoid the development of adverse reactions such as drowsiness, muscular relaxation, and electrolyte disturbances [62]. A Malaysian randomized control study data revealed that a loading dose of 200 milligrams per kilogram of magnesium sulfate was necessary for the treatment modality to work in PPHN [62]. This research found a significant enhancement in oxygenation and reduced the demand for ventilatory support when magnesium sulfate was used compared to the control group [62]. Nebulized magnesium sulfate was demonstrated to be an effective therapeutic intervention in 28 term neonates with PPHN, as revealed in a study conducted in India in the year 2020 [62].

*Thromboxane Synthase Inhibito*rs

According to a study conducted in Japan in 1994, thromboxane A2 (TXA2) was proven to constrict arteries and bronchial airways, predisposing patients of PPHN to aggravating breathlessness [63]. The randomized control trial that was carried out on 78 patients not only explained the role of platelet activation in enhancing the TXA2 production but also showcased the importance of TXA2 synthase inhibitors in curbing the symptoms of breathlessness in patients with established PPHN [64]. In another case-controlled study conducted on animals at the outset of hypoxia, furegrelate sodium showed a significant reduction by 34% in the vascular impedance, thereby proving the importance of TXA2 synthase blocker itself [65].

Endothelin Receptor Blockers

Endothelin-1 (ET-1), a potent vascular modulator that is derived from peptides, tends to interact with endothelin and its receptors which are found on pulmonary smooth muscles [66]. Endothelins are derived from peptides. The endothelin receptors are found upon the lung’s smooth muscles and the isopeptide that interacts [66]. ET-1 is a potent vasoconstrictor, and concentrations of it in the embryonic circulations are elevated. It works by blocking both the endothelium sites. In the case reports by Radicioni et al., it is as effective as a supplement to iNO and oral sildenafil [66]. The conclusion of a case study that focused on the endothelin receptor blocker in a newborn with PPHN indicated that bosentan might When more traditional therapies fail in the life-threatening PPHN, it might be used as a supplement [66]. According to a case study conducted in Thailand, an oral double ET-1 blocker called Bosentan proved to be an efficacious management modality in patients of PPHN with previously failed treatments [67].

Inhaled Nitrous Oxide

Nitric oxide synthetase (NOS) acts on the nitrogen ions of arginine, which results in the production of NO in the vascular endothelial cells [68,69]. Although NOS has other pre-existing isoforms inside the airway, the endothelium variant of NOS is an essential regulator of NO generation, allowing its dispersion and action on endothelium and smooth muscles, respectively [70]. The messenger ribonucleic acid and proteins for pulmonary endothelium NOS are available in early fetal life and rise with the growing gestational age of the fetus, thereby preparing it for adaption outside the womb [69]. Term-infants tend to have a stronger reaction to iNO than preterm infants [69].

Newborn infants at/or around term had a stronger reaction to endothelium active vasodilators including iNO, oxygen, or acetylcholine than all those born preterm [68,69]. The cGMP gated signal transduction is activated by cGMP [70]. These channels block the extracellular influx of calcium by activating calcium-sensitive potassium channels, resulting in transmembrane polarization and decreased smooth vascular strength [70].

 iNO tends to operate locally without causing systemic effects as it binds with hemoglobin to create nitrosyl hemoglobin which is converted to methemoglobin after entering the body [71]. In term newborns, iNO was demonstrated to help improve the need for oxygen and minimize the necessity for ECMO [71,72]. According to a systematic review, after the administration of iNO, a rise of 53 millimeters of mercury (mmHg) and a drop of 15.1 minutes was noticed in the partial pressure of oxygen (PaO_2_) [73]. The iNO administration should be begun at 20 parts per million (ppm) with careful monitoring of methemoglobin and NO concentrations in the patient [74,75]. A randomized trial in term newborns, starting iNO at 15%-25% instead of values higher than 25%, improved the aeration process but did not reduce the ECMO or mortality [76].

iNO administration has shown to have a positive impact on the aeration of infants diagnosed with CDH, thereby improving management modalities in patients with an increased predisposition for developing PPHN [77]. Retrospective research revealed that iNO administration through the nasal cannula might assist a subpopulation of CDH babies with late pulmonary arterial hypertension [78]. A review article published by Nicolas et al. in the year 2012 concluded that early rescue therapy of preterm born children with iNO based on aeration criteria failed to show any meaningful impact on death or borderline personality disorder in nine studies [78]. Despite this evidence, a systematic assessment of 155,872 children born around 34 weeks gestation treated between the years 2000 and 2008 instituted that the overall iNO usage rose from 0.3% to 1.8% and 0.8% to 7% in babies birth during 23 to 26 weeks, respectively [79].

High-Frequency Oscillation (HFO)

Despite the increased need for HFO and its ability to enhance respiration and carbon dioxide removal in newborns with PPHN, there has not been any significant evidence recommending its usage as a management modality for the disease [80]. A randomized trial that was conducted in Georgia, United States of America, aimed to compare the safety and efficacy of high-frequency oscillation with conventional ventilation in the treatment of neonates with respiratory failure, where they had a data sample of 79 patients where 40 were assigned to traditional ventilation and 39 to HFO [80]. The study revealed that only 44% of the conventional ventilation group were responsive compared to 63% in the high-frequency oscillation group. This led to the conclusion that HFO is a safe and effective rescue technique in treating neonates with respiratory failure [80]. According to a research study conducted on patients with severe PPHN, the use of HFO was contrasted with the administration of iNO and mechanical ventilators [81]. Newborns who resisted the initial treatment with a poor increase in their oxygenation were given an alternative management modality to curb their symptoms [81]. Multicenter randomized control trials are being carried out since HFO has not proven beneficial in a good number of infants suffering from PPHN [82].

Extracorporeal Membrane Oxygenation

A meta-analysis of four ECMO studies in babies with acute respiratory failure found that ECMO was linked with substantially increased survival, with a relative risk of 0.82 [83]. The ECMO research group published a one-to-seven-year follow-up which revealed that ECMO assistance lowered mortality risk without increasing the chance of severe disability [84]. Moreover, the study implicated in the UK highlighted the severity of the disease since more than half of the children had died or were severely crippled by the age of seven [84]. ECMO results have varied over the years depending upon the underlying etiology, with a 90% survival rate observed in infants diagnosed with MAS [85]. On the other hand, 78% of newborns with BPD showed continued existence with an uninterrupted life support reliance and neurodevelopmental adversities [85].

A study conducted on 73 children diagnosed with CDH who used ECMO from 1991 to 2000 tracked their improvement until 2003 [85]. It was revealed that 37% showed a mere one-year survival rate [85]. At the follow-up, 48% of survivors had lung issues, 59% had gastrointestinal symptoms, and 19% had severe neurological deficits [85]. About 12% of those who survived seemed to have no substantial neurobehavioral imbalance and did not require further medical-surgical involvement [85].

Limitations

Potential alternates to the traditional treatment modalities have all been taken into account. However, in-depth trials on their efficacy and efficiency still need to be conducted to provide solid alternatives and a structured treatment plan of action.

## Conclusions

In conclusion, PPHN is a common newborn disease characterized by oxygenation failure, but it encompasses a broad range of physiological factors that must be addressed when making treatment choices. According to the findings from the studies above, when contemplating alternate pulmonary vasodilators or medications to promote proper ventricular function, the latter is significant from the studies reviewed as these drugs are readily available and are associated with better outcomes. Although used as the first-line treatment modality, oxygen therapy should be administered cautiously as the risk of developing complications is high. Despite breakthroughs in the treatment of newborns with PPHN in recent.
